# Supplementary material for: Individual retrotransposon integrants are differentially controlled by KZFP/KAP1-dependent histone methylation, DNA methylation and TET-mediated hydroxymethylation in naïve embryonic stem cells
Source: Epigenetics Chromatin. 2018 Feb 26;11:7. doi: 10.1186/s13072-018-0177-1 (PMC6389204; doi:10.1186/s13072-018-0177-1)
Supplement: Supplementary file 11 — Additional file 11. Pattern analysis. [file 13072_2018_177_MOESM11_ESM.zip › Patterns analysis/DataTables/extensions/Scroller/examples/server-side_processing.html]

Scroller example - Server-side processing (5,000,000 rows)


# Scroller example Server-side processing (5,000,000 rows)

DataTables' server-side processing mode is a feature that naturally fits in with Scroller perfectly.
Server-side processing can be used to show large data sets, with the server being used to do the data
processing, and Scroller optimising the display of the data in a scrolling viewport.

When using server-side processing, Scroller will wait a small amount of time to allow the scrolling
to finish before requesting more data from the server (200mS by default). This prevents you from DoSing
your own server!

This example shows Scroller using server-side processing mode and 5 million rows.
**Important** This particular example uses `ajaxDT` as a function to 'fake' the data to
show Scroller's ability to show large data sets. It does not have a real database behind it! You would
normally not use `ajaxDT` as a function to generate data, but
rather as a url for where to fetch the real data!

In this example we also enable the `loadingIndicator` option of Scroller to show the end
user what is happening when they scroll passed the currently loaded data.

| ID | First name | Last name | ZIP / Post code | Country |
| --- | --- | --- | --- | --- |

- Javascript
- HTML
- CSS
- Ajax
- Server-side script

The Javascript shown below is used to initialise the table shown in this
example:

`$(document).ready(function() {
$('#example').DataTable( {
serverSide: true,
ordering: false,
searching: false,
ajax: function ( data, callback, settings ) {
var out = [];
for ( var i=data.start, ien=data.start+data.length ; i<ien ; i++ ) {
out.push( [ i+'-1', i+'-2', i+'-3', i+'-4', i+'-5' ] );
}
setTimeout( function () {
callback( {
draw: data.draw,
data: out,
recordsTotal: 5000000,
recordsFiltered: 5000000
} );
}, 50 );
},
dom: "rtiS",
scrollY: 200,
scroller: {
loadingIndicator: true
}
} );
} );`

In addition to the above code, the following Javascript library files are loaded for use in this
example:

- ../../../media/js/jquery.js
- ../../../media/js/jquery.dataTables.js
- ../js/dataTables.scroller.js

The HTML shown below is the raw HTML table element, before it has been enhanced by
DataTables:

This example uses a little bit of additional CSS beyond what is loaded from the library
files (below), in order to correctly display the table. The additional CSS used is shown
below:

The following CSS library files are loaded for use in this example to provide the styling of the
table:

- ../../../media/css/jquery.dataTables.css
- ../css/dataTables.scroller.css

This table loads data by Ajax. The latest data that has been loaded is shown below. This data
will update automatically as any additional data is loaded.

The script used to perform the server-side processing for this table is shown below. Please note
that this is just an example script using PHP. Server-side processing scripts can be written in any
language, using the protocol described in the
DataTables documentation.

## Other examples

### Examples

- Basic initialisation
- State saving
- Client-side data source (50,000 rows)
- Server-side processing
  (5,000,000 rows)
- API

Please refer to the DataTables documentation for full
information about its API properties and methods.  
Additionally, there are a wide range of extras and
plug-ins which extend the capabilities of
DataTables.

DataTables designed and created by SpryMedia Ltd © 2007-2014  
DataTables is licensed under the MIT license.
